# Stabilization and optimization of host-microbe-environment interactions as a potential reason for the behavior of natal philopatry

**DOI:** 10.1186/s42523-021-00087-3

**Published:** 2021-03-30

**Authors:** Ting-bei Bo, Kevin D. Kohl

**Affiliations:** 1 State Key Laboratory of Integrated Management of Pest Insects and Rodents, Institute of Zoology, Chinese Academy of Sciences, Beijing, 100101 China; 2 CAS Center for Excellence in Biotic Interactions, University of Chinese Academy of Sciences, Beijing, 100049 China; 3 Department of Biological Sciences, University of Pittsburgh, Pittsburgh, PA USA

## Abstract

Many animals engage in a behavior known as natal philopatry, where after sexual maturity they return to their own birthplaces for subsequent reproduction. There are many proposed ultimate factors that may underlie the evolution of natal philopatry, such as genetic optimization, suitable living conditions, and friendly neighbors, which can improve the survival rates of offspring. However, here we propose that a key factor that has been overlooked could be the colonization of gut microbiota during early life and the effects these microorganisms have on host performance and fitness. In addition to the bacteria transmitted from the mother to offspring, microbes from the surrounding environment also account for a large proportion of the developing gut microbiome. While it was long believed that microbial species all have global distributions, we now know that there are substantial geographic differences and dispersal limitations to environmental microbes. The establishment of gut microbiota during early life has enormous impacts on animal development, including energy metabolism, training of the immune system, and cognitive development. Moreover, these microbial effects scale to influence animal performance and fitness, raising the possibility for natural selection to act on the integrated combination of gut microbial communities and host genetics (i.e. the holobiont). Therefore, in this paper, we propose a hypothesis: that optimization of host-microbe-environment interactions represents a potentially important yet overlooked reason for natal philopatry. Microbiota obtained by natal philopatry could help animals adapt to the environment and improve the survival rates of their young. We propose future directions to test these ideas, and the implications that this hypothesis has for our understanding of host-microbe interactions.

## Introduction

In nature, many species with long migration distances display some degree of natal philopatry, or returning to their birthplace to breed the next generation [1, 2]. By means of this activity, animals can meet the environmental conditions they need during a specific period of life, likely resulting in higher survival and fitness of individuals [3–5]. However, the ultimate mechanisms underlying the evolution of natal philopatry are still poorly understood. There has been a recently renewed appreciation for the role that host-associated microbes play in the performance and fitness of wild animals [6–9], including aspects of animal behaviors [10]. In this perspective article, we propose a potential role for these interactions to play in the evolution of natal philopatry. Specifically, we argue that by parents returning to natal habitats to breed, new offspring will acquire the optimal microbiome for their own physiological performance and fitness. We recognize that this idea is largely speculative but feel that it represents an exciting area for future research.

Natal philopatry is a life-history strategy where animals locate and return to reproduce at the same geographic location in which they were born [11]. An equivalent term, “natal homing”, was first used when describing the migratory and breeding habits of sea turtles [12]. In addition to sea turtles and well-known groups such as songbirds and salmon, many other animal groups engage in natal philopatry (Table 1). The prevalence and taxonomic distribution of natal philopatry has been reviewed elsewhere for mammals [30] and birds [31, 32].

**Table 1:** Examples of natal philopatry

	**Insects** -- The spotted darter dragonfly (*Sympetrum depressiusculum*) exhibits strong philopatry to their natal ponds [13]. -- The lesser marbled fritillary butterfly (*Brenthis ino*) returns to natal sites [14].
	**Fish** -- Numerous species of salmonids (salmon, trout, char, etc.) migrate back to natal sites for spawning [15]. -- In marine weakfish (*Cynoscion regalis*), spawning site fidelity ranges from 60 to 81% [16]. --- Several shark species exhibit philopatric behavior [17].
	**Amphibians** --In marbled salamanders (*Ambystoma opacum*), roughly 90% of individuals return to their natal ponds for breeding [18]. --Several anuran species (frogs and toads) exhibit strong philopatry to their natal ponds [19–21].
	**Reptiles** -- Both male and female green sea turtle (*Chelonia mydas*) return to natal rookeries to breed [22, 23]. -- Female pine snakes (*Pituophis melanoleucus*) repeatedly return to nesting sites [24].
	**Birds** -- Great Reed Warblers (*Acrocephalus arundinaceus*) exhibit limited dispersal, and remain in their natal habitats [25]. -- The Wandering Albatross (*Diomedea exulans*) exhibits high philopatry, where 70-92% of individuals breed at natal sites [26].
	**Mammals** -- Bottlenose dolphins (*Tursiops truncatus*) return to breed in their natal habitats well into adulthood [27]. -- In the brown long-eared bat (*Plecotus auritus*), both sexes exhibit philopatry to natal roosts [28]. -- Female Antarctic fur seals (*Arctocephalus gazella*) return to breed within a few meters of their own birth site [29].

In recent decades, there has been a renewed appreciation for the fact that animals evolved in a world already dominated by microbes [33]. Therefore, many physiological processes in animals are intertwined with the action of microbial symbionts. Host-associated microbial communities have a number of impacts on their hosts, such as assisting with digestion or nutrient synthesis [34], providing protection against pathogens [35], aiding in the development of the immune system [36], and determining life history traits [37]. The hologenomic theory of evolution considers the collection of host and microbial genomes—known as ‘the holobiont’—a biological unit of organization upon which natural selection can act [38, 39]. There are numerous examples of connections between hologenomic evolution and aspects of animal behavior [10]. Here, we propose that microorganisms present in the natal habitat may be more suitable or beneficial for offspring, and thus returning to the natal habitat for reproduction may provide a fitness benefit to animals. We set up the rationale for this hypothesis by first reviewing the basics of natal philopatry, and then discussing how host-microbe interactions fit within these existing theories.

## Mechanisms for Natal Philopatry

How do animals locate their birthplaces to reproduce and generate offspring? Several proximate mechanisms for natal philopatry have been proposed. Early work on homing in turtles hypothesized that this behavior was proximately driven through “social facilitation”, where first-time breeders follow experienced females to a nesting beach, and having had a “favorable” breeding experience, fix on that site for future nesting [2, 40]. Related, Nordeng et al. (1977) proposed a “pheromone hypothesis”, where anadromous salmon might produce population-specific odors which guide them in homing migration [41]. However, imprinting on the natal site itself is seems to be the most accepted mechanism underlying this behavior. Genetic analysis of natal philopatry in sea turtles is quite consistent with natal homing expectations and indicate that social facilitation to non-natal sites is uncommon [42]. There is some evidence sea turtles may imprint on the geomagnetic features of their natal beaches to return to these sites for reproduction [43]. Additionally, although definitive evidence is lacking, it is widely assumed that Pacific salmon imprint on key features of their nesting region, like the chemical profile of the beach or surrounding waters, during development, and then use this information to return as adults [44].

While these hypotheses explain the proximate mechanisms of how animals are able to locate their natal sites, the evolutionary reasons, or ultimate mechanisms, for natal philopatry are still poorly understood. Below we briefly outline the current theories of the ultimate mechanisms for natal philopatry. For example, the advantage of local knowledge may enable animals to optimally exploit the resources of the area and to successfully defend their territories against competitors [45, 46]. Thus, over many generations and through natural selection, populations would become adapted to the conditions prevailing in their natal habitat [45, 46]. Indeed, individual Collard Flycatchers that are more philopatric exhibit higher reproductive fitness than those that disperse over larger distances [47]. Similarly, philopatric individuals of the Great Reed Warbler exhibit higher lifetime fitness when compared to immigrant individuals [5].

## Reasons for animal’s natal philopatry

### Evolutionary and genetic optimization

The distance that young animals disperse from their place of origin before breeding has important implications for the extent of inbreeding and for the genetic structure of populations. Indeed, natal philopatry tends to result in high relatedness among individuals at a particular site [48, 49]. It has been proposed that natal philopatry may actually promote an optimal rate of inbreeding. While inbreeding is often viewed as detrimental due to the increased fixation of deleterious alleles, it can also offer benefits if alleles at various loci across the genome interact with each other in a beneficial manner. Thus, optimal inbreeding can reduce the costs of meiosis and recombination by preserving these interactions [50]. Such genetic optimization could be especially important for local adaptation associated with philopatry. For example, Coho salmon (*Oncorhynchus kisutch*) from more interior streams have greater swimming stamina as an adaptation to the longer migration distances [51]. It has previously been argued that gene flow between populations may erode these local adaptations [52]. Further, given that complex traits and adaptations are likely polygenic, maintenance of interacting alleles could also be important [53]. These genetic mechanisms could underlie the evolutionary benefits of natal homing behaviors.

### Suitable environmental factors

Animals may also return to their birthplace for reproduction because these natal environments are most suitable for the growth and development of their young, including suitable temperature, abundant food resources, low abundances of predators, and nesting sites with superior geographical location. For example, female Pine snakes (*Pituophis melanoleucus*) show a high degree of nest site philopatry because it promotes location of sites that provide suitable temperature conditions [54]. Temperate zone bat species are believed to form summer maternity colonies to provide the appropriate thermal conditions for the growth and survival of their offspring [55]. Natal philopatry in bannertailed kangaroo rats (*Dipodomys spectabilis*) may be a means of providing juveniles with access to essential resources, such as food caches and large complex burrow systems, that are not readily available outside natal home ranges [56]. Overall, by returning to the same site for reproduction each year, individuals can be assured of the suitable environmental conditions to support growth, maturation, and survival of their young.

### Maintaining social networks

Natal homing can help animals establish familiar community structure and reduce resource competition. In Antarctic fur seals (*Arctocephalus gazella*), adults of both sexes return to within a few meters of the breeding sites that were held in previous years [57]. Such behavior could potentially be adaptive in a crowded and highly competitive environment, perhaps by facilitating the re-occupation of previously held territories [58] or by creating stable neighborhood networks in which overall levels of conflict are minimized [59].

Beyond reducing competition, natal philopatry may help to maintain familiar social networks. For bottlenose dolphins (*Tursiops truncatus*), philopatry offers the benefits of familiar social networks and foraging habitats [60]. Additionally, it is thought that natal philopatry in Brown long-eared bats (*Plecotus auritus*) could be driven by the social benefits of associating with familiar individuals [28]. Thus, homing behavior may serve as a mechanism to sort and distribute animals, and in the process may maintain social benefits or decrease intraspecific competition for resources.

## Another potential reason for natal philopatry: host-microbe-environment interactions

The fact that animals evolved in a world already dominated by microbes [33] opens the possibility for microbial involvement in the processes of natal philopatry. First, microbes could play a role in the proximate mechanisms of animals locating their natal sites. While many animals imprint on the geomagnetic features of their natal sites [43], we have a poor understanding of how animals sense and respond to magnetic fields, including the fact that bona fide animal magnetoreceptors have still not been identified [61]. It has recently been proposed that symbiotic magnetotactic bacteria could contribute to these capabilities [62]. For example, host-associated magnetotactic bacteria may aggregate based on the geomagnetic field and provide signals to their hosts [62]. Additionally, microbes may be involved in the location of natal sites using chemical cues given the large repertoire of volatile compounds they produce, which are also involved in inter-kingdom interactions [63]. For example, microbes create volatile compounds that may be used for inter-host communication, such as on the scent glands of hyenas [64] or the uropygial glands of birds [65]. Some soil bacteria also generate volatile compounds that attract small invertebrates (springtails), which then feed on the bacteria and mediate the dispersal of bacterial spores [66] . Therefore, it could be that local environmental microbes produce volatile compounds to act as proximate signals for the location of natal sites.

Beyond the location of natal sites, we propose that acquiring the optimal composition and structure of microorganisms may also be a reason why animals return to their birthplace for reproduction. Analogous to the ultimate factors previously proposed for the evolution of natal philopatry (Evolutionary and genetic optimization, Suitable environmental factors, and Maintaining social networks), we propose that host-microbe interactions during early life could be involved in (i) evolutionary and hologenomic optimization, (ii) acclimating to local environmental conditions and (iii) the social benefits of natal philopatry. While the connections between natal philopatry and microbial ecology have not been thoroughly investigated, we present a series of studies that provide interesting data supporting this hypothesis.

### Evolutionary and hologenomic optimization

Our understanding of the evolutionary importance of host-microbe interactions is rapidly increasing. Host-microbe interactions have the capacity to affect many aspects of host performance and fitness [67]. Numerous studies across animal taxa have demonstrated that there are optimal combinations between host genetics and associated microbial communities, such that inoculating animals with the microbial communities from heterologous host species reduces performance and fitness [9, 68–70]. For example, individuals of *Peromyscus polionotus* inoculated with the microbes from other Peromyscus species exhibit decreased rates of food digestibility [9]. Additionally, both *Drosophila* flies, *Nasonia* wasps, and *Caenorhabditis* worms exhibit decreased fitness when inoculated with the microbiomes of congener hosts [9, 68–70]. Last, animals that are the hybrids of two species often suffer from incompatibilities between the host genome and the microbiome, leading to detrimental effects, such as gut inflammation or lethality [71, 72]. Thus, it is increasingly being recognized that we must expand our view of biological interactions to be a combination of Genome_Host_ x Genome_Microbial_ x Environment interactions [38, 73]. For wild animals, ensuring that offspring associate with the proper microbial community in early life (described more in the sections below) could have large fitness consequences for reproducing animals.

These microbial associations can be especially important during early life [74]. Animals exhibit developmental windows during which microbial interactions are especially crucial [75]. For example, disruption of the microbiome in larval zebrafish can cause lasting changes to behavior and neurodevelopment [76]. Similarly, tadpoles reared under depleted microbial conditions exhibit increased susceptibility to later parasitic [77] and viral infections [78]. Moreover, early associations with particular microbes in early life can also influence the ability for subsequent microbes to take hold, a process known as priority effects or historical contingency [79]. Therefore, exposure to early environmental microbes could have life-long impacts on what other microbes are able to colonize animals. Given the effects that microbes can have on host digestion [34], protection against pathogens [35], the development of the immune system [36], associating with the proper microbial communities in early life has the potential to yield lifelong fitness effects.

### Microbes as environmental factors

How then, do animals obtain their microbiome? Starting at birth or hatching, the microbiota develops from a simple, unstable community into a complex and climax community [74]. While a portion of the microbiome is vertically transmitted [80], a number of environmental microbes are also important early colonizers [81]. For example, some animal species predominantly acquire host-associated microbial communities from the environment each new generation, such as stinkbugs [82] and bioluminescent squid [83]. Studies in tadpoles [84] and several fish species [85, 86] demonstrate that the gut microbiota largely reflects those microbes present in their surrounding environments. In nest-building species, such as birds, the characteristics and environmental microbiome of nesting material can influence the composition of their offspring’s microbiome [87, 88]. Experimental research on laboratory mice [89] and wild *Peromyscus* mice [90] have also demonstrated that the gut microbiome of mammals can be determined by their juvenile environment. Thus, the microbiome of the surrounding environment could have important implications for the assembly of host-associated communities.

Additionally, environmental microbial populations exhibit substantial spatial heterogeneity across the landscape. While it had long been assumed that “everything is everywhere” [91], recent studies demonstrate that both terrestrial and aquatic microbial communities exhibit considerable biogeographic trends, resulting in heterogeneity of microbial diversity and community structure over geographic space [92, 93]. This spatial heterogeneity may contribute to the well-documented effects of geography on host-associated microbial communities [94]. For example, when comparing the gut microbial communities wild mice obtained from across France and Germany, results showed that patterns of microbiota diversity were principally explained by the geographical location of the mice, with weaker effects due to the genetic distance [95]. Other work has found that sympatric populations of gorillas and chimpanzees share more bacterial taxa than gorillas and chimps from disparate regions, suggesting a potential of limits to microbial dispersal [96]. The cloacal microbiota of fledgling greater flamingos (*Phoenicopterus roseus*) varied substantially across nine breeding sites, suggesting that local environments harbor distinct microbial communities [97].

The geographic variation in environmental microbes may carry “signatures” relating to characteristics of the local environment. For example, over large geographic scales the biogeographic distribution of soil microbes is driven by temperature [98, 99]. Host-microbe interactions have been implicated in thermal physiology of hosts [100] and their adaptations to local climate [101]. Thus, it could be that animals acquire environmental microbes that are adapted to certain aspects of the local environment (temperature, frequency of disturbance, etc.), and thereby convey related physiological benefits to their hosts. However, it should be noted that we still have a poor understanding of the spatial heterogeneity of environmental microbes on spatial scales that may be ecologically relevant to natal philopatry. For example, in a study of philopatric songbirds, a dispersal distance of roughly 1000 m from the natal habitat yielded lower fitness [47], while studies investigating site-specific avian microbial communities typically use greater spatial distances between sites [97, 102].

### Microbes as a social benefit

Analogous to how natal philopatry may yield the social benefits through cooperation or reduced competition, there may be microbial benefits to living together [103]. The behavior of allo-coprophagy, where offspring consume the feces of adult individuals, including unrelated individuals, has been recorded in insects [104], reptiles [105, 106], birds [107], and mammals [108, 109]. This behavior can be important for juveniles to become inoculated with a microbial community. For example, cockroaches regularly consume feces of conspecifics, and doing so results in colonization of the gut and enhanced growth and tissue development [110] . Similarly, green iguanas regularly consume the feces of adult individuals [105], and doing so increases their ability to digest plant material and improves growth rates [111].

Even if not a principal source of microbial inoculation, group living has also been demonstrated to result in microbial sharing between individuals. Numerous social or gregarious insect species transmit some beneficial microbes between individuals [112–114]. In baboons, the degree of social interactions between individuals can explain a significant portion of microbial sharing [115]. Additionally, dispersal of individuals to join unrelated groups can result in acquisition of new microbial communities, and individuals that engage in more grooming behavior share similar microbial communities [116]. Similarly, affiliative behaviors between unrelated individuals of feral horses correlate with similarities in gut microbial community structure [117]. Thus, returning to the natal site for breeding may expose offspring to a social network and associated microbial symbionts that may be acquired through social transmission.

## Caveats

It is important to acknowledge that our framework also has caveats. First, while natal philopatry is a relatively common behavior across animal taxa (Table 1), studies of this behavior may suffer from a reporting bias towards those that exhibit philopatry or are easier to study [32]. Second, natal philopatry is not always an adaptive behavior. In contrast to studies cited earlier, a study of Western gulls showed that philopatric individuals had lower survival and reproductive fitness, leading to a conclusion that the benefits of this life history strategy may vary temporally and spatially [118]. Similarly, the benefits of microbial interactions may also be variable across space and time. Related, our framework is largely rationalized by philopatry promoting the stabilization of environment-host-microbe interactions. However, novel microbial communities are thought to be a route by which animals may gain enhanced capabilities and adapt to novel environments [119]. Therefore, there may be some benefits to animals dispersing to new habitats for reproduction, which may lead to the acquisition of novel microbes in offspring. Such benefits could underlie the observed variability in the degree of site fidelity across individuals. Overall, more studies are needed to test ideas of our framework in a broad array of systems.

## Future directions

**Fig. 1:**
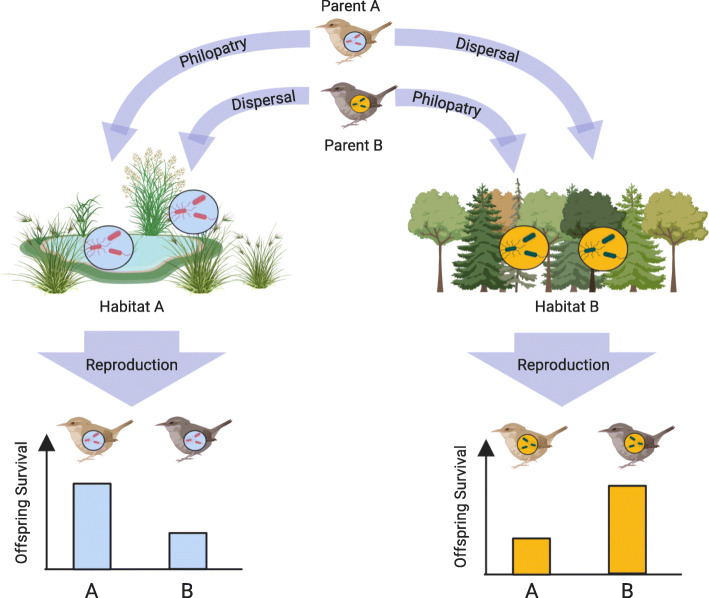
Diagrammatic representation of how host-microbe interactions may influence reproductive success of philopatric or dispersing individuals. Individuals that exhibit philopatry maintain optimal Genome_Host_ x Genome_Microbial_ x Environment interactions. Conversely, individuals that disperse experience incompatibility in Genome_Host_ x Genome_Microbial_ x Environment interactions, and suffer fitness consequences

With these research findings and caveats in mind, we propose a conceptual framework by which environmental acquisition of microbes and associated fitness benefits may contribute to the evolution of natal philopatry (Fig. 1). Here, individuals that exhibit philopatry return to their birthplace for reproduction, and thus their offspring acquire the optimal microbial communities during early life and exhibit improved performance and survival. Conversely, offspring of individuals that had dispersed for reproduction have a mismatch between host genetics and microbial genetics, and the environment. Such sub-optimal matching may result in decreased performance and fitness of offspring in these environments. Such fitness consequences may yield evolution in behavioral traits, such as natal homing, especially since natural selection is thought to act strongly on behavior [120], and behavioral traits are evolutionarily labile [121]. Through these processes, host-microbe interactions and the resulting fitness benefits could act as ultimate factors underlying the evolution of natal philopatry.

However, we again recognize that our framework is highly speculative. Future observational, comparative, and experimental work could help to address these ideas and large open questions that remain in this framework. First, a thorough understanding of the basic microbial ecology of animal systems that engage in natal philopatry is still lacking. Overall, microbiome research has been largely biased towards model systems and biomedical studies [122–124]. In many wild systems we still lack thorough knowledge on the routes of microbial acquisition, microbial sharing, and the effects of microbial communities on performance and fitness. Therefore, researchers should employ current techniques for inventorying the taxonomic composition and functions of microbial communities [125] and integrating these techniques with questions of integrative and comparative biology [125, 126]. Additionally, as mentioned above, we have poor understanding of the resolution of spatial heterogeneity in microbial communities, especially on scales relevant to animal ecology.

Another large question relates to the concept Genome_Host_ x Genome_Microbial_ x Environment interactions, and which of these interactions are most important in determining performance and fitness. Classical work to test Genome x Environment interactions, such as common garden or transplant experiments, have largely conflated the aspects of Genome_Host_ and Genome_Microbial_. Additionally, existing work on Genome_Host_ x Genome_Microbial_ interactions have largely ignored environmental variables, as they have been conducted in captivity under constant conditions [9, 68–70]. Thus, to our knowledge, full tests of Genome_Host_ x Genome_Microbial_ x Environment interactions remain lacking. In the future, a combination of controlled microbial inoculations during early life followed by exposure to varying environments would benefit the framework presented in this article, as well as the greater field of host-microbe interactions.

We predict that manipulative experiments will provide powerful approaches to disentangle the effects of genetics, location, and the microbiome on breeding success. Some groups have used cross-fostering approaches in nestling birds to demonstrate that the local environment determines the membership of their gut microbiome [127]. Similar approaches could be taken in philopatric species. Additionally, controlled experimental studies in the laboratory could be useful. Several studies have manipulated environmental microbial communities in the lab to investigate their effects on animal performance [77, 128]. Similar approaches could be used with multiple environmental microbial sources. Following such microbial manipulations, researchers could release animals into experimental field sites and track performance and fitness. For example, researchers have used controlled environmental mesocosms to track the population dynamics of released lizards [129], voles [130], fish [131] and invertebrates [132]. Combining experimental microbial manipulations with mesocosm approaches will be a necessary next step in testing aspects of our conceptual framework.

Overall, it is becoming more and more clear that microbial partnerships influence many aspects of animal life-history and revealing a role for these partnerships in the enigmatic behavior of natal philopatry could have numerous implications. First, testing whether microbial interactions play a role in the evolution of natal philopatry will greatly enhance our understanding of hologenomic evolution [38], including the evolution of animal behavior [10] . Additionally, understanding the proximate and ultimate causes of natal philopatry has conservation implications to ensure successful breeding [13, 133]. Similarly, host-microbe interactions have the potential to influence conservation efforts [8], and thus understanding the role of microbes in early life success could help to shape and prioritize breeding sites and captive breeding efforts.

## Conclusion

This article highlights the potential for host-microbe interactions to underly the evolution of natal philopatry. Numerous studies have demonstrated the importance of environmental microbes in colonizing animal hosts, and the effect of specific host-microbe associations on animal performance. Thus, we argue that the combination of these factors may lead animals to return to natal sites for breeding to ensure that their offspring acquire the optimal microbiome.

## Data Availability

Not applicable.

## References

[1] Cava JA, Perlut NG, Travis SE (2016). Why come back home? Investigating the proximate factors that influence natal philopatry in migratory passerines. Anim Behav.

[2] Hendrickson JR (1958). The green sea turtle, Chelonia mydas (Linn.) in Malaya and Sarawak. Proc Zool Soc London.

[3] Forschler MI, Val ED, Bairlein F (2010). Extraordinary high natal philopatry in a migratory passerine. J Ornithol.

[4] Kristensen TN, Loeschcke V, Hoffmann AA (2008). Linking inbreeding effects in captive populations with fitness in the wild: release of replicated *Drosophila melanogaster* lines under different temperatures. Conserv Biol.

[5] Bensch S, Hasselquist D, Nielsen B, Hansson B (1998). Higher fitness for philopatric than for immigrant males in a semi-isolated population of great reed warblers. Evolution..

[6] Waite DW, Taylor MW (2014). Characterizing the avian gut microbiota: membership, driving influences, and potential function. Front Microbiol.

[7] Kohl KD (2012). Diversity and function of the avian gut microbiota. J Comp Physiol B.

[8] Trevelline BK, Fontaine SS, Hartup BK, Kohl KD (2019). Conservation biology needs a microbial renaissance: a call for the consideration of host-associated microbiota in wildlife management practices. Proc R Soc B.

[9] Brooks AW, Kohl KD, Brucker RM, van Opstal EJ, Bordenstein SR (2016). Phylosymbiosis: relationships and functional effects of microbial communities across host evolutionary history. PLoS Biol.

[10] Theis KR, Whittaker DJ, Rojas CA, Banzhaf W, BHC C, Deb K, Holekamp KE, Lenski RE, Ofria C, Pennock RT, Punch WF, Whittaker DJ (2020). A hologenomic approach to animal behavior. In: evolution in action: past, present and future: a festschrift in honor of Erik D Goodman.

[11] Papi F (2006). Navigation of marine, freshwater and coastal animals: concepts and current problems. Mar Freshw Behav Physiol.

[12] Ehrenfeld DW, Carr A. The role of vision in the sea-finding orientation of the green turtle (*Chelonia mydas*). Anim Behav. 1967;15(1):25–36. 10.1016/s0003-3472(67)80007-1 6031107

[13] Dolný A, Mižicova H, Harabis F (2013). Natal philopatry in four European species of dragonflies (*Odonata: Sympetrinae*) and possible implications for conservation management. J Insect Conserv.

[14] Weyer J, Schmitt T (2013). Knowing the way home: strong philopatry of a highly mobile insect species. Brenthis ino J Insect Conserv.

[15] Keefer ML, Caudill CC (2014). Homing and straying by anadromous salmonids: a review of mechanisms and rates. Rev Fish Biol Fish.

[16] Thorrold SR, Latkoczy C, Swart PK, Jones CM (2001). Natal homing in a marine fish metapopulation. Science..

[17] Hueter RE, Heupel MR (2004). Evidence of philopatry in sharks and implications for the management of shark fisheries. J Northwest Atl Fish Sci.

[18] Gamble LR, Mcgarigal K, Compton BW (2007). Fidelity and dispersal in the pond-breeding amphibian, Ambystoma opacum: implications for spatio-temporal population dynamics and conservation. Biol Conserv.

[19] Berven KA, Grudzien TA (1990). Dispersal in the wood frog (*Rana Sylvatica*): implications for genetic population structure. Evolution..

[20] Breden F (1987). The effect of post-metamorphic dispersal on the population genetic structure of Fowler's toad, *Bufo woodhousei fowleri*. Copeia..

[21] Reading CJ, Loman J, Madsen T (1991). Breeding pond fidelity in the common toad, *Bufo bufo*. J Zool.

[22] Fitzsimmons NN, Limpus CJ, Norman JA, Goldizen AR, Miller JD, Moritz C (1997). Philopatry of male marine turtles inferred from mitochondrial DNA markers. PNAS..

[23] Lee PLM, Luschi P, Hays GC (2006). Detecting female precise natal philopatry in green turtles using assignment methods. Mol Ecol.

[24] Burger J, Zappalorti R (1992). Philopatry and nesting phenology of pine snakes *Pituophis melanoleucus* in the New Jersey pine barrens. Behav Ecol Sociobiol.

[25] Hansson B, Bensch S, Hasselquist D, Nielsen B (2002). Restricted dispersal in a long-distance migrant bird with patchy distribution, the great reed warbler. Oecologia..

[26] Gauthier G, Milot E, Weimerskirch H (2010). Small-scale dispersal and survival in a long-lived seabird, the wandering albatross. J Anim Ecol.

[27] Mchugh K, Allen J, Barleycorn AAA, Wells RS (2011). Natal philopatry, ranging behavior, and habitat selection of juvenile bottlenose dolphins in Sarasota Bay. Florida J Mammal.

[28] Burland T, Barratt E, Nichols R, Racey PA (2001). Mating patterns, relatedness and the basis of natal philopatry in the brown long-eared bat, *Plecotus auritus*. Mol Ecol.

[29] Hoffman JI, Forcada J (2012). Extreme natal philopatry in female Antarctic fur seals (*Arctocephalus gazella*). Mamm Biol.

[30] Waser PM, Jones WT (1983). Natal philopatry among solitary mammals. Q Rev Biol.

[31] Coulson JC (1938). A review of philopatry in seabirds and comparisons with other waterbird species. Waterbirds..

[32] Weatherhead PJ, Forbes MRL (1994). Natal philopatry in passerine birds: genetic or ecological influences?. Behav Ecol.

[33] Mcfall-Ngai M, Hadfield MG, Bosch TCG, Carey HV, Domazet-loso T, Douglas AE (2013). Animals in a bacterial world, a new imperative for the life sciences. PNAS.

[34] Cummings JH, Macfarlane GT (1997). Role of intestinal bacteria in nutrient metabolism. Clin Nutr.

[35] Fukuda S, Toh H, Hase K, Oshima K, Nakanishi Y, Yoshimura K (2011). *Bifidobacteria* can protect from enteropathogenic infection through production of acetate. Nature..

[36] Round JL, Mazmanian SK (2009). The gut microbiota shapes intestinal immune responses during health and disease. Nat Rev Immunol.

[37] Sisonmangus MP, Mushegian AA, Ebert D (2015). Water fleas require microbiota for survival, growth and reproduction. ISME J.

[38] Bordenstein SR, Theis KR. Host biology in light of the microbiome: ten principles of holobionts and hologenomes. PLoS Biol. 2015;13(8):e1002226. 10.1371/journal.pbio.1002226 PMC4540581 26284777

[39] Zilber-Rosenberg I, Rosenberg E (2008). Role of microorganisms in the evolution of animals and plants: the hologenome theory of evolution. FEMS Microbiol Rev.

[40] Owens DW, Grassman MA, Hendrickson JR (1982). The imprinting hypotheses and sea turtle reproduction. Herpetolog- ica.

[41] Nordeng H (1977). A pheromone hypothesis for homeward migration in anadromous salmonids. Oikos..

[42] Meylan AB, Bowen BW, Avise JC (1990). A genetic test of the natal homing versus social facilitation models for green turtle migration. Science..

[43] Lohmann KJ (2015). Evidence for geomagnetic imprinting and magnetic navigation in the natal homing of sea turtles. Curr Biol.

[44] Dittman AH, Quinn TP (1996). Homing in pacific salmon: mechanisms and ecological basis. J Exp Biol.

[45] Greenwood PJ, Harvey PH (1982). The natal and breeding dispersal of birds. Annu Rev Ecol Syst.

[46] Newton I (2008). The migration ecology of birds.

[47] Part T (1991). Philopatry pays: a comparison between collared flycatcher sisters. Am Nat.

[48] Nichols HJ, Cant MA, Hoffman JI, Sanderson J (2014). Evidence for frequent incest in a cooperatively breeding mammal. Biol Lett.

[49] Rueger T, Harrison HB, Buston PM, Gardiner NM, Berumen ML, Jones GP. Natal philopatry increases relatedness within groups of coral reef cardinalfish. Proc R Soc B. 2020;287(1930):20201133. 10.1098/rspb.2020.1133 PMC7423479 32635871

[50] Shields WM. Philopatry, inbreeding, and the evolution of sex: Suny Press; 1982.

[51] Taylor JM, Mcphail JD (1985). Burst swimming and size-related predation of newly emerged coho salmon *Oncorhynchus kisutch trans*. Am Fish Soc.

[52] Tigano A, Friesen VL (2016). Genomics of local adaptation with gene flow. Mol Ecol.

[53] Akerman A, Bürger R (2014). The consequences of gene flow for local adaptation and differentiation: a two-locus two-deme model. J Math Biol.

[54] Burger J, Zappalorti RT (1992). Philopatry and nesting phenology of pine snakes *Pituophis melanoleucus* in the New Jersey pine barrens. Behav Ecol Sociobiol.

[55] Mcnab BK (1982). Ecology of bats.

[56] Jones WT (1984). Natal philopatry in bannertailed kangaroo rats. Behav Ecol Sociobiol.

[57] Forcada J, Trathan PN, Murphy EJ (2008). Life history buffering in Antarctic mammals and birds against changing patterns of climate and environmental variation. Glob Chang Biol.

[58] Forstmeier W (2002). Benefits of early arrival at breeding grounds vary between males. J Anim Ecol.

[59] Beletsky LD, Orians GH (1989). Familiar neighbours enhance breeding success in birds. Proc Natl Acad Sci.

[60] Tsai YJ, Mann J (2013). Dispersal, philopatry, and the role of fission-fusion dynamics in bottlenose dolphins. Mar Mamm Sci.

[61] Wiltschko R, Wiltschko W (2019). Magnetoreception in birds. J R Soc Interface.

[62] Natan E, Fitak RR, Werber Y, Vortman Y (2020). Symbiotic magnetic sensing: raising evidence and beyond. Philos T R Soc B.

[63] Kristin SB, Martín-Sánchez L, Paolina G (2017). Microbial volatiles: small molecules with an important role in intra- and inter-kingdom interactions. Front Microbiol.

[64] Theis KR, Venkataraman A, Dycus JA, Koonter KD, Schmitt-Matzen EN, Wagner AP, Holekamp KE, Schmidt TM. Symbiotic bacteria appear to mediate hyena social odors. Proc Natl Acad Sci. 2013;110(49):19832–7. 10.1073/pnas.1306477110 PMC3856825 24218592

[65] Whittaker DJ, Slowinski SP, Greenberg JM, Alian OM (2019). Experimental evidence that symbiotic bacteria produce chemical cues in a songbird. J Exp Biol.

[66] Becher PG, Verschut V, Bibb MJ, Bush MJ, Molnar BP, Barane E (2020). Developmentally regulated volatiles geosmin and 2-methylisoborneol attract a soil arthropod to Streptomyces bacteria promoting spore dispersal. Nat Microbiol.

[67] Gould AL, Zhang V, Lamberti L, Jones EW, Obadia B, Korasidis N (2018). Microbiome interactions shape host fitness. PNAS..

[68] Adair KL, Bost A, Bueno E, Kaunisto S, Kortet R, Peters-Schulze G (2020). Host determinants of among-species variation in microbiome composition in drosophilid flies. ISME J.

[69] Berg M, Zhou XY, Shapira M (2016). Host-specific functional significance of *Caenorhabditis* gut commensals. Front Microbiol.

[70] van Opstal EJ, Bordenstein SR (2019). Phylosymbiosis impacts adaptive traits in *Nasonia* wasps. mBio.

[71] Brucker RM, Bordenstein SR (2013). The hologenomic basis of speciation: gut bacteria cause hybrid lethality in the genus Nasonia. Science..

[72] Wang J, Kalyan S, Steck N, Turner LM, Harr B, Kunzel S (2015). Analysis of intestinal microbiota in hybrid house mice reveals evolutionary divergence in a vertebrate hologenome. Nat Commun.

[73] Carrier TJ, Reitzel AM. The hologenome across environments and the implications of a host-associated microbial repertoire. Front Microbiol. 2017;8:802. 10.3389/fmicb.2017.00802 PMC5425589 28553264

[74] Tamburini S, Shen N, Wu HC, Clemente JC (2016). The microbiome in early life: implications for health outcomes. Nat Med.

[75] Cox LM, Yamanishi S, Sohn J, Alekseyenko AV, Leung JM, Cho I (2014). Altering the intestinal microbiota during a critical developmental window has lasting metabolic consequences. Cell..

[76] Phelps D, Brinkman NE, Keely SP, Anneken EM, Catron TR, Betancourt D (2017). Microbial colonization is required for normal neurobehavioral development in zebrafish. Sci Rep.

[77] Knutie SA, Wilkinson CL, Kohl KD, Rohr JR (2017). Early-life disruption of amphibian microbiota decreases later-life resistance to parasites. Nat Commun.

[78] Warne RW, Kirschman LJ, Zeglin LH (2019). Manipulation of gut microbiota during critical developmental windows affects host physiological performance and disease susceptibility across ontogeny. J Anim Ecol.

[79] Martinez I, Maldonadogomez MX, Gomesneto JC, Kittana H, Ding H, Schmaltz R, et al. Experimental evaluation of the importance of colonization history in early-life gut microbiota assembly. eLife. 2018;7. 10.7554/eLife.36521. 10.7554/eLife.36521 PMC6143339 30226190

[80] Funkhouser LJ, Bordenstein SR. Mom knows best: the universality of maternal microbial transmission. PLoS Biol. 2013;11(8):e1001631. 10.1371/journal.pbio.1001631 PMC3747981 23976878

[81] Dominguez-Bello MG, Costello EK, Contreras M, Magris M, Hidalgo G, Fierer N (2010). Delivery mode shapes the acquisition and structure of the initial microbiota across multiple body habitats in newborns. PNAS..

[82] Kikuchi Y, Hosokawa T, Fukatsu T (2007). Insect-microbe mutualism without vertical transmission: a stinkbug acquires a beneficial gut symbiont from the environment every generation. Appl Environ Microbiol.

[83] Nyholm SV, Mcfallngai MJ (2004). The winnowing: establishing the squid– vibrio symbiosis. Nat Rev Microbiol.

[84] Correa DT, Rodriguez D, Emer C, Saenz D, Adams CK, Schiesari L, Matz M, Leibold MA. Multilevel community assembly of the tadpole gut. bioRxiv. 2020. 10.1101/2020.07.05.188698.

[85] Shangong W, Esther RA, Wang W, Li W, Zou H (2012). Composition, diversity, and origin of the bacterial community in grass carp intestine. PLoS One.

[86] Wong S, Stephens WZ, Burns AR, Stagaman K, David LA, Bohannan BJM, et al. Ontogenetic differences in dietary fat influence microbiota assembly in the zebrafish gut. Mbio. 2015;6(5):e00687–15. 10.1128/mBio.00687-15 PMC4611033 26419876

[87] Campos-Cerda F, Bohannan BJ (2020). The Nidobiome: a framework for understanding microbiome assembly in neonates. Trends Ecol Evol.

[88] Ruiz-Rodríguez M, Martín-Vivaldi M, Martínez-Bueno M, Soler JJ (2018). Gut microbiota of great spotted cuckoo nestlings is a mixture of those of their foster magpie siblings and of cuckoo adults. Genes..

[89] Snijders AM, Langley SA, Kim YM, Brislawn CJ, Noecker C, Zink EM (2017). Influence of early life exposure, host genetics and diet on the mouse gut microbiome and metabolome. Nat Microbiol.

[90] Schmidt E, Mykytczuk NCS, Schultehostedde AI (2019). Effects of the captive and wild environment on diversity of the gut microbiome of deer mice (*Peromyscus maniculatus*). ISME J.

[91] Beijerinck MJM, Amsterdam. De infusies en de ontdekking der backteriën. Jaarboek van de Koninklijke Akademie voor Wetenschappen. 1913.

[92] Hellweger FL, van Sebille E, Fredrick ND (2014). Biogeographic patterns in ocean microbes emerge in a neutral agent-based model. Science..

[93] Fierer N, Jackson RB (2006). The diversity and biogeography of soil bacterial communities. PNAS..

[94] Moeller AH, Suzuki TA, Lin D, Lacey EA, Wasser SK, Nachman MW (2017). Dispersal limitation promotes the diversification of the mammalian gut microbiota. PNAS..

[95] Weldon L, Abolins S, Lenzi L, Bourne C, Riley EM, Viney MJ. The gut microbiota of wild mice. PLoS One. 2015;10(8):e0134643. 10.1371/journal.pone.0134643 PMC4530874 26258484

[96] Moeller AH, Ndjango JB, Li Y, Hahn BH, Ochman H (2013). Sympatric chimpanzees andgorillas harbor convergent gut microbial communities. Genome Res.

[97] Gillingham MAF, Béchet A, Cézilly F, Wilhelm K, Rendón-Martos M, Borghesi F (2019). Offspring microbiomes differ across breeding sites in a panmictic species. Front Microbiol.

[98] Garcia-Pichel F, Loza V, Marusenko Y, Mateo P, Potrafka RM (2013). Temperature drives the continental-scale distribution of key microbes in topsoil communities. Science.

[99] Nottingham AT, Noah F, Turner BL, Whitaker J, Ostle NJ, McNamara NP (2018). Microbes follow Humboldt: temperature drives plant and soil microbial diversity patterns from the Amazon to the Andes. Ecology.

[100] Moeller AH, Ivey K, Cornwall MB, Herr K, Rede J, Taylor EN, et al. Lizard gut microbiome changes with temperature and is associated with heat tolerance. Appl Environ Microbiol. 2020;86(17):e01181–20. 10.1128/AEM.01181-20 PMC7440792 32591376

[101] Suzuki TA, Martins FM, Phiferixey M, Nachman MW. The gut microbiota and Bergmann's rule in wild house mice. Mol Ecol. 2020;29(12):2300–11. 10.1111/mec.15476 PMC8073627 32419280

[102] Kreisinger J, Kropackova L, Petrželkova A, Adamkova MK, Tomasek O, Martin J, et al. Temporal stability and the effect of transgenerational transfer on fecal microbiota structure in a long distance migratory bird. Front Microbiol. 2017;8. 10.3389/fmicb.2017.00050. 10.3389/fmicb.2017.00050 PMC5292904 28220109

[103] Archie EA, Tung J (2015). Social behavior and the microbiome. Curr Opin Behav Sci.

[104] Korner M, Diehl JMC, Meunier J (2016). Growing up with feces: benefits of Allo-coprophagy in families of the European earwig. Behav Ecol.

[105] Troyer K (1984). Behavioral acquisition of the hindgut fermentation system by hatchling *Iguana iguana*. Behav Ecol Sociobiol.

[106] Vicenzi N (2015). *Phymaturus palluma* (high mountain lizard) coprophagy. Herpetol Rev.

[107] Kobayashi A, Tsuchida S, Ueda A, Yamada T, Murata K, Nakamura H (2019). Role of coprophagy in the cecal microbiome development of an herbivorous bird Japanese rock ptarmigan. J Vet Med Sci.

[108] Crowelldavis SL, Houpt KA (1985). Coprophagy by foals: effect of age and possible functions. Equine Vet J.

[109] Osawa R, Blanshard W, Ocallaghan P (1993). Microbiological studies of the intestinal microflora of the koala, phascolarctos-cinereus. 2. Pap, a special maternal feces consumed by juvenile koalas. Aust J Zool.

[110] Jahnes BC, Herrmann M, Sabree ZL (2019). Conspecific coprophagy stimulates normal development in a germ-free model invertebrate. PeerJ..

[111] Troyer K (1982). Transfer of fermentative microbes between generations in a herbivorous lizard. Science..

[112] Koch H, Schmidhempel P (2011). Socially transmitted gut microbiota protect bumble bees against an intestinal parasite. PNAS..

[113] Martinez AJ, Ingham CS, Kaltenpoth M, Onchuru TO (2018). Transmission of mutualistic bacteria in social and gregarious insects. Curr Opin Insect Sci.

[114] Salem H, Florez LV, Gerardo NM, Kaltenpoth M (2015). An out-of-body experience: the extracellular dimension for the transmission of mutualistic bacteria in insects. Proc R Soc B.

[115] Tung J, Barreiro LB, Burns MB, Grenier JC, Lynch J, Grieneisen LE, et al. Social networks predict gut microbiome composition in wild baboons. eLife. 2015;4(4):e05224. 10.7554/eLife.05224 PMC4379495 25774601

[116] Grieneisen LE, Livermore J, Alberts SC, Tung J, Archie EA (2017). Group living and male dispersal predict the core gut microbiome in wild baboons. Integr Comp Biol.

[117] Antwis RE, Lea JMD, Unwin B, Shultz S (2018). Gut microbiome composition is associated with spatial structuring and social interactions in semi-feral Welsh Mountain ponies. Microbiome..

[118] Spear LB, Pyle P, Nur N (1998). Natal dispersal in the western gull: proximal factors and fitness consequences. J Anim Ecol.

[119] Alberdi A, Aizpurua O, Bohmann K, Zepeda-Mendoza ML, Gilbert M (2016). Do vertebrate gut metagenomes confer rapid ecological adaptation?. Trends Ecol Evol.

[120] Boulding EG (2010). Experimental evolution. Concepts, methods, and applications of selection experiments. Theodore Garland Jr and Michael R. rose, editors. Integr Comp Biol.

[121] Blomberg SP, Garland JR, Ives AR (2003). Testing for phylogenetic signal in comparative data: behavioral traits are more labile. Evolution..

[122] Colston TJ, Jackson CR (2016). Microbiome evolution along divergent branches of the vertebrate tree of life: what is known and unknown. Mol Ecol.

[123] Greysongaito CJ, Bartley TJ, Cottenie K, Jarvis WMC, Newman AEM, Stothart MR (2020). Into the wild: microbiome transplant studies need broader ecological reality. Proc R Soc B.

[124] Pascoe EL, Hauffe HC, Marchesi JR, Perkins SE (2017). Network analysis of gut microbiota literature: an overview of the research landscape in non-human animal studies. ISME J.

[125] Liu YX, Qin Y, Chen T, Lu M, Qian X, Guo X, et al. A practical guide to amplicon and metagenomic analysis of microbiome data. Protein Cell. 2020:1–16. 10.1007/s13238-020-00724-8. 10.1007/s13238-020-00724-8 PMC8106563 32394199

[126] Kohl KD (2017). An introductory "how-to" guide for incorporating microbiome research into integrative and comparative biology. Integr Comp Biol.

[127] Teyssier A, Lens L, Matthysen E, White J. Dynamics of gut microbiota diversity during the early development of an avian host: evidence from a cross-foster experiment. Front Microbiol. 2018;9. 10.3389/fmicb.2018.01524. 10.3389/fmicb.2018.01524 PMC6046450 30038608

[128] van Veelen HJ, Salles JF, Matson KD, van der Velde M, Tieleman BI (2020). Microbial environment shapes immune function and cloacal microbiota dynamics in zebra finches Taeniopygia guttata. Anim Microbio.

[129] Bestion E, Jacob S, Zinger L, Gesu LD, Richard M, White JL (2017). Climate warming reduces gut microbiota diversity in a vertebrate ectotherm. Nat Ecol Evol.

[130] Forbes KM, Henttonen H, Hirvelakoski V, Kipar A, Mappes T, Stuart P (2015). Food provisioning alters infection dynamics in populations of a wild rodent. Proc R Soc B.

[131] Keller AA, Kleinmacphee G (2000). Impact of elevated temperature on the growth, survival, and trophic dynamics of winter flounder larvae: a mesocosm study. Can J Fish Aquat Sci.

[132] Wieczorek MV, Bakanov N, Bilancia D, Szocs E, Stehle S, Bundschuh M (2018). Structural and functional effects of a short-term pyrethroid pulse exposure on invertebrates in outdoor stream mesocosms. Sci Total Environ.

[133] Kruk M, Noordervliet MAW, Keurs WJT (1998). Natal philopatry in the black-tailed godwit *Limosa limosa* L. and its possible implications for conservation. Ringing Migr.

